# Effect of bio-polyol molecular weight on the structure and properties of polyurethane-polyisocyanurate (PUR-PIR) foams

**DOI:** 10.1038/s41598-023-50764-3

**Published:** 2024-01-08

**Authors:** Adam Olszewski, Paulina Kosmela, Laima Vēvere, Mikelis Kirpluks, Ugis Cabulis, Łukasz Piszczyk

**Affiliations:** 1 Department of Polymer Technology, Chemical Faculty, Gdansk University of Technology, G. Narutowicza St. 11/12, 80-233 Gdansk, Poland; 2 Advanced Materials Center, Gdańsk University of Technology, Gabriela Narutowicza 11/12, 80-233 Gdańsk, Poland; 3 Polymer Laboratory, Latvian State Institute of Wood Chemistry, Dzerbenes 27, Riga, 1006 Latvia

**Keywords:** Structural materials, Materials science, Porous materials

## Abstract

The increasing interest in polyurethane materials has raised the question of the environmental impact of these materials. For this reason, the scientists aim to find an extremely difficult balance between new material technologies and sustainable development. This work attempts to validate the possibility of replacing petrochemical polyols with previously synthesized bio-polyols and their impact on the structure and properties of rigid polyurethane-polyisocyanurate (PUR-PIR). To date, biobased polyols were frequently used in the manufacturing of PU, but application of bio-polyols synthesized via solvothermal liquefaction using different chains of polyethylene glycol has not been comprehensively discussed. In this work, ten sets of rigid polyurethane foams were synthesized. The influence of bio-polyols addition on foam properties was investigated by mechanical testing, thermogravimetric analysis (TGA), and cone calorimetry. The structure was determined by scanning electron microscopy (SEM) and a gas pycnometer. The tests revealed a significant extension of foam growth time, which can be explained by possible steric hindrances and the presence of less reactive secondary hydroxyl groups. Moreover, an increase average size of pores and aspect ratio was noticed. This can be interpreted by the modification of the cell growth process by the introduction of a less reactive bio-polyol with different viscosity. The analysis of foams mechanical properties showed that the normalized compressive strength increased up to 40% due to incorporation of more cross-linked structures. The thermogravimetric analysis demonstrated that the addition of bio-based polyols increased temperature of 2% (T_2%_) and 5% (T_5%_) mass degradation. On the other hand, evaluation of flammability of manufactured foams showed increase of total heat release (HRR) and smoke release (TSR) what may be caused by reduction of char layer stability. These findings add substantially to our understanding of the incorporation of bio-polyols into industrial polyurethane systems and suggest the necessity of conducting further research on these materials.

## Introduction

Since the development of the first industrial techniques for the production of polymers and plastics, these materials have changed the outlook of materials science and civilizational progress. Today, plastics are an integral part of our daily lives, and their properties make them almost irreplaceable. The multitude of types of plastics and their processing provide a wide spectrum of applications for these materials^1^. One of the most widely used polymeric materials are polyurethanes (PU), which structure and properties can be precisely designed and easily changed^2^. This provides a great versatility for these materials that can be used in the building and construction industry, automotive sector, packaging, clothing industry, furniture industry and others^3^.

The synthesis of PU materials involves a polyaddition reaction between di- and poly-isocyanates and compounds containing at least two hydrogen-donating hydroxyl groups, which leads to the generation of urethane groups in the growth of the polymer chain^4^. In addition to these substances, polyurethane materials also consist of catalysts, low-molecular weight chain extenders, blowing agents, surfactants, fillers, and many other additives, which give the final properties of the material^5,6^. The proper choice of components allows for the manufacturing of PU materials such as rigid resins^7^, reactive adhesives^8^, PU coatings and thin layers^9^ and polyurethane foams^10,11^. In the case of polyurethane foams, these materials can be divided by their structure (close or open cell structures), behavior (soft, semi-rigid, rigid) or density. Furthermore, a strong relationship between the structure/properties of the foam and possible industrial application is widely discussed in the literature^12^.

This worldwide interest in PU has raised the question of the environmental performance of such materials^13^. Unfortunately, PU materials are mostly composed of petrochemical resources which large-scale use leads to fossil fuels depletion, environmental pollution, and greenhouse gas emissions^14^. For this reason, industrial companies and scientists should aim to find an extremely difficult balance between new material technologies and sustainable development. One of the most popular trends to reduce the use of nonrenewable resources in PU material is the replacement of petrochemical polyols with bio-polyols, which are obtained from renewable raw materials. The most important substances for bio-polyol production are cellulose^15^, lignin^16^, castor oil^17^, rapeseed oil^18^, soybean oil^19^, mustard seed oil^20^, algae^21^, wood bark and wood processing wastes^8^. Such a replacement process provides not only environmental benefits, but can also contribute to the improvement of PU quality, material price, and post-use waste management due to increased biodegradability.

To date scientists developed many processes, which allow for the transformation of natural origin substances into polyols. One of the most interesting processes for the PU industry may be solvothermal liquefaction^22^. The process involves a solvolysis reaction between solvents and biomass which leads to the cleavage of biomass chains into lower molecular weight compounds that contain a high amount of hydroxyl groups. This process occurs in the temperature range of 120–250 °C under normal pressure^13^. To accelerate the process, usually up to 3% addition of a homogeneous catalyst is used. As a homogenous catalyst, strong acids (e.g., sulphuric acid^23^; p-toluenesulfonic acid^24^) and strong bases (e.g., sodium hydroxide and potassium hydroxides)^25^ may be used. The main product of this reaction may contain a mixture of compounds rich in hydroxyl groups. These compounds may include glycerol derivatives (e.g., condensed glycerol), glycols, esters, ethers, carbohydrates, and carboxylic acids. Moreover, the synthesized product may contain a significant amount of water which could be removed by the process of drying under reduced pressure. After drying and the purification process, the obtained product may be used as a substitute of petrochemical polyols during PU manufacturing. The most commonly manufactured materials with the addition of this type of polyol are PU foams^26^, but the area of PU adhesives^8^ and composites^27^ is attracting considerable interest.

Zhang et al.^28^ performed an optimalization of biomass liquefaction of four agricultural wastes—wheat straw (WS), corn stover (CS), rice straw (RS), and oilseed rape straw (OS). The authors used a mixture of ethylene glycol and polyethylene glycol 400 (PEG400). As a catalyst, sulfuric acid was used. The authors provide a detailed analysis of reaction temperature, reaction time, liquid and solid ratio, the ratio between PEG and EG, straw particle size, and catalyst dosage on parameters of bio-polyols. The properties of polyols have been studied by using biomass conversion ratio, hydroxyl value titration, rheological studies, Fourier transform infrared spectroscopy (FTIR) and two-dimensional correlation spectroscopy (2D-COS). The authors concluded that temperature and catalyst concentration have a greater influence on bio-polyol properties parameters than other above-mentioned parameters. Synthesized polyols were used as substitutes for petrochemical polyol in PU foam composition. Manufactured foams had properties that allow their potential use in industry.

The work of Hu et. al.^29^ offers an analysis of the feasibility of crude glycerol in the solvothermal liquefaction of soybean straw for the synthesis of polyols which can be used for PU foam manufacturing. Authors concluded that reaction conditions at 240 °C, time > 180 min, and 3% addition of catalyst provide polyols with a combination of the most favorable properties (LOH in range from 440 to 540 mg KOH/g, viscosities from 16 to 45 Pa, and acid numbers below 5 mg KOH/g). Selected polyols were used for the manufacturing of PU foams. Depending on the density measurement and compressive strength, the authors concluded that the properties of the obtained polyols and foams are comparable to those obtained using petroleum-based compounds.

The study by Lu et al.^30^ shows the possibility of the use of hardwood residue (by-product of the paper industry) in the solvothermal liquefaction process in the presence of polyethylene glycol 400 (PEG400), ethylene carbonate (EC) and sulfuric acid. Authors suggested possible reaction mechanisms and examined properties of bio-polyols by FTIR, determination of residue content, acid number and hydroxyl number titration. The reaction conducted at a temperature of 160 °C, the reaction time of 60 min, the ratio of PEG400/EC of 8:2 (w/w), and the ratio of liquid/solid of 5:1 (w/w) provided polyols with preferable properties which were used for PU foam manufacturing. Authors claim that the replacement of petrochemical polyol caused an increase in apparent density and compressive strength of the polyurethane foams. This effect decreases with the addition of polyol above 5%.

Considering available scientific literature, bio-based polyols synthesized via biomass liquefaction are successfully used for PU materials manufacturing. Moreover, it can be noticed that these polyols significantly influence the properties of PU materials. For this reason, scientists should focus on the relationship between the parameters of solvothermal liquefaction and the properties of the PU materials. To date, most research on the replacement of petrochemical polyol with substances synthesized by solvothermal liquefaction has focused on the use of various types of biomasses and solvents, including polyethylene glycols. Despite interest, previous studies have failed to compare the properties of materials manufactured using bio-polyols obtained using various molecular weight PEGs. It should be emphasized that in our previous research^31^, it was indicated that polyols synthesized using PEG with molecular mass in the range from 200 to 600 g/mol have different hydroxyl values, degree of biomass conversion, molecular weight, viscosity, and thermal stability. Each of these parameters may have a huge impact on the structure and final properties of designed materials.

Summarizing, as the properties of bio-based polyols may have a significant influence on the structure and properties of PU, this work examines the influence of three polyols synthesized via biomass liquefaction using polyethylene glycols with different molecular masses on the properties of rigid PU foams. To validate this hypothesis, ten sets of PU rigid foams with the addition of bio-polyols using one-step method were synthesized. The foaming process of rigid PU foams was characterized by a universal foam qualification system Foamat. The properties of manufactured materials were examined by mechanical testing, thermogravimetric analysis (TGA), cone calorimetry and thermal conductivity measurement. Moreover, the structure of the foams was characterized using a gas pycnometer and scanning electron microscopy (SEM). The results of this study will greatly contribute to the understanding of bio-polyols influence on the structure and properties of rigid PU foams and will provide information if synthesized polyols can be industrially applicable.

## Experimental

### Materials

The list of applied components with all necessary details is presented in Table 1.

**Table 1:** Components for manufacturing of rigid foams.

Substrate	Producer	Properties/additional information
Elapol 8020	ELAchem Spa (Corso Torino, Italy)	HV = 250 mg KOH/g; Viscosity at 25 °C = 3.5–4.5 Pa∙s; %_H2O_ < 0.15%; pH ≈ 7.0;
Glycerol	Merck (Darmstadt, Germany)	HV = 1200 mg KOH/g; Viscosity at 20 °C = 1.5 Pa s;
P_200	^31^	HV = 652 mg KOH/g; BC = 94.3%; Viscosity at 30 °C = 0.736 Pa∙s; %_H2O_ = 0.82; M_n_ = 3837 g/mol; pH ≈ 6.3;
P_400	^31^	HV = 519 mg KOH/g; BC = 91.4%; Viscosity at 30 °C = 0.790 Pa∙s; %_H2O_ = 0.67; M_n_ = 4365 g/mol; pH ≈ 6.6;
P_600	^31^	HV = 589 mg KOH/g; BC = 90.9%; Viscosity at 30 °C = 1.415 Pa∙s; %_H2O_ = 0.64; M_n_ = 4536 g/mol; pH ≈ 6.5;
PMDTA	Merck (Darmstadt, Germany)	N,N,N′,N′′,N′′- Pentamethyldiethylenetriamine, catalyst
Dabco K15	Air Products (Allentown, Pennsylvania, U.S.)	75% wt potassium octoate solution in diethylene glycol, catalyst
PC CAT® TKA30	Performance Chemicals (Belvedere. United Kingdom)	33 wt% potassium acetate solution in ethylene glycol, catalyst;
DABCO TMR 7	Air Products (Allentown, Pennsylvania, U.S.)	An amine carboxylate-based trimer catalyst;
Tegostab B 8465 (SPC)	Evonik Industries AG (Essen, Germany)	Silicone surfactant;
TCPP	LANXESS Deutschland GmbH (Cologne, Germany)	Tris(chloropropyl) phosphate, fire retardant;
Solkane® 365/227	Solvay (Brussel, Belgium)	Blend of 1,1,1,3,3-pentafluorobutane and 1,1,1,2,3,3,3-heptafluoropropane, foaming agent;
Polymeric 4,4′-methylene diphenyl diisocyanate (pMDI)	BASF (Mannheim, Germany)	31.5% content of NCO groups;

*HV* hydroxyl value, *%*_*H2O*_ water content, *BC* biomass conversion, *M*_*n*_ average molecular weight.

### Preparation of rigid polyurethane foams

Rigid foams were manufactured by the one-step method using two-component system (A and B). Component A was composed of the petrochemical polyol Elapol 8020, catalysts, foaming agents, surfactant, flame retardant and 0–30% addition of three bio-polyols synthesized in our previous research^31^. Bio-polyols were synthesized via biomass liquefaction of cellulose in a mixture of glycerol and PEGs with different molecular masses (PEG 200/PEG 400/PEG 600). Component A was premixed at 1000 rpm for 30 s to provide a homogeneous mixture of components. Component B was composed of commercially available polymeric 4,4′-methylene diphenyl diisocyanate (pMDI). Isocyanate index (the ratio of NCO–OH groups) was determined according to the calculation of the chemical equivalent of isocyanate and hydroxyl groups and was equal to 3. Both components were mixed at 2000 rpm for 10 s in a plastic cup and poured into a mold (200 × 200 × 150 mm). Manufactured polyurethane foams were conditioned for 24 h at room temperature. The reference sample without bio-polyols was coded P_0. Samples with bio-polyol addition were coded P_XXX_YY, where XXX indicates the type of bio-polyol and YY amount of polyol. The composition of manufactured rigid polyurethane foams is presented in Table 2. The amount of foaming agent was modified each time to obtain foams with comparable density (32 ± 2 kg/m^3^). It should be noted that the proposed formulations were designed by modification of foam formulations which are used in the industry.

**Table 2:** Formulations to synthesize 100g of each polyurethane foams.

Compoment	P_0	P_200_10	P_200_20	P_200_30	P_400_10	P_400_20	P_400_30	P_600_10	P_600_20	P_600_30
	Component mass [g]									
Elapol 8020	31.67	26.07	21.35	17.32	26.81	22.50	18.65	27.48	22.70	18.56
P_200	x	2.90	5.34	7.42	x	x	x	x	x	x
P_400	x	x	x	x	2.98	5.63	7.99	x	x	x
P_600	x	x	x	x	x	x	x	3.05	5.68	7.95
Glycerol	0.49	0.44	0.41	0.38	0.46	0.43	0.41	0.47	0.44	0.41
PMDTA	0.14	0.13	0.12	0.11	0.13	0.13	0.12	0.14	0.13	0.12
Dabco K15	0.60	0.55	0.51	0.47	0.57	0.54	0.51	0.58	0.54	0.51
PC CAT® TKA30	0.17	0.15	0.14	0.13	0.16	0.15	0.14	0.16	0.15	0.14
DABCO TMR 7	0.15	0.14	0.13	0.12	0.15	0.14	0.13	0.15	0.14	0.13
SPC	1.02	0.93	0.86	0.80	0.96	0.91	0.86	0.98	0.91	0.85
TCPP	2.03	1.86	1.71	1.58	1.91	1.80	1.71	1.96	1.82	1.70
Solkane® 365/227	5.08	4.64	4.28	3.97	4.78	4.51	4.27	4.89	4.55	4.25
pMDI	58.65	62.18	65.15	67.69	61.10	63.28	65.22	60.13	62.95	65.39

### Characterization

#### Evaluation of the foaming process

The evaluation of the foaming process (n ≥ 3) was performed using the universal foam qualification system FOAMAT® 285 (Format-Messtechnik GmbH, Freiburg im Breisgau, Germany). During the measurement, start time, rise time, foam growth rate and shrinkage were measured. The results were used to determine the effect of bio-polyols on the reactivity of the system.

#### Apparent density

The apparent density of manufactured bio-based rigid polyurethane foams was estimated in accordance with PN-EN ISO 845:2010.

#### Open cell content

The open/closed cell content was determined using a gas pycnometer Ultrapyc 5000 Foam manufactured by Anton Paar (Graz, Austria). The samples (n ≥ 3) were analyzed in the following settings: gas–nitrogen; target pressure—3.0 psi; measurement type—corrected; temperature control—on; target temperature—20.0 °C; flow mode—monolith; preparation mode—flow for 2 min.

#### Microstructure of PU foams

The structure of manufactured PUR-PIR foams was determined using scanning electron microscopy (SEM). The test was carried out on a microscope Hitachi FlexSEM 1000II (Tokyo, Japan). SEM images of the foams were taken at a magnification of 70 × and 10 kV electron beam acceleration voltage. Foam cells parameters were analyzed using Image J software (n ≤ 25).

#### Compressive strength

The compressive strength of the manufactured materials in the perpendicular and parallel directions was determined by a uniaxial compression test based on the PN-EN ISO 844:2021-09. Compression tests were performed at room temperature on a Zwick/Roell 1000 N testing machine (Zwick GmbH & Co, Ulm, Germany) at a constant speed of 10%/min until the deformation reached 30%. The dimensions of the cylindric samples (n ≥ 5). were 20 × 20 mm (height and diameter).

#### Tensile strength

The tensile strength of the rigid polyurethane foams was determined by a uniaxial tensile test on a Zwick/Roell Z010 TN universal testing machine (Zwick GmbH & Co, Ulm, Germany) at a constant speed of 10 mm/min until failure (n ≥ 5). The dimensions of dumbbell-shaped samples were: gauge length—100 mm, thickness—10 mm, width—15 mm.

#### Thermal conductivity coefficient

The thermal conductivity coefficient (λ) of PUR-PIR foams was determined using the Laser Comp Heat Flow Instrument Fox 200 (New Castle, DE, USA) according to ISO 8301. Foams (n ≥ 3). were tested 24 h after manufacture. The temperature difference between plates was 20 °C (heated plate T_HP_ = 20 °C and cold plate T_CP_ = 0 °C). The tested samples had dimensions of 200 mm × 200 mm × 30 mm.

#### Thermogravimetric analysis

The thermal stability of the PUR-PIR foams was determined by thermogravimetric analysis (TGA). These tests were carried out using the NETZSCH TG 209 F3 apparatus (NETZSCH-Gruppe, Selb, Germany). The measurements of 10 ± 1 mg samples were made in the temperature range of 30–800 °C and at a heating rate of 10 °C/min in a nitrogen-constant flow.

#### Fire behavior of PU foams

The influence of bio-polyols on the combustion process was determined according to ISO 5660 using a cone calorimeter from Fire Testing Technology Ltd. (East Grinstead, UK). PUR-PIR foams (n ≥ 3) with the dimensions of 100 × 100 × 30 mm were placed 25 mm away from the cone heater and exposed horizontally to an external heat flux of 35 kW/m^2^. TTI—time to ignition, TTF—time to flameout, THR—total heat release, pHRR—maximum peak of heat release rate, AvHRR—average heat release rate, MAHRE—maximum average rate of heat emission, TSR—total smoke release; TSP—total smoke production; AvCO—average CO emission, AvCO_2_—average CO_2_ emission were determined during this test.

## Results and discussion

### Foaming process of rigid polyurethane foams modified by bio-polyols

**Figure 1:**
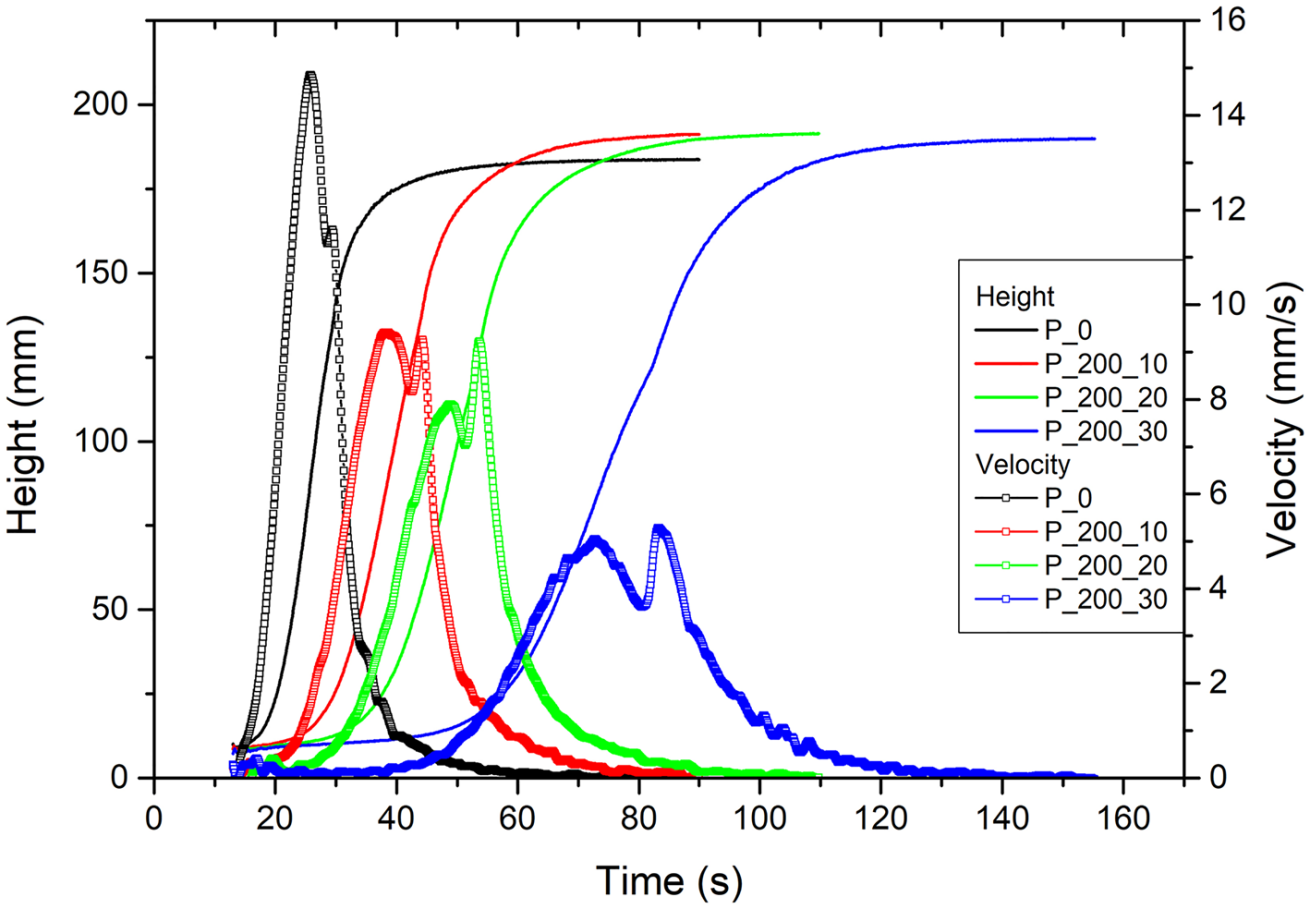
Foaming process of samples with addition of P_200 polyols.

As each component of polyurethane material composition has a significant influence on the foaming process and cellular structure, the influence of previously synthesized polyols was determined using FOAMAT device. Details of the foaming process of synthesized polyurethane foams are shown in Table 3 and Fig. 1.

**Table 3:** Parameters of foaming process of synthesized polyurethane foams.

Foam symbol	Rise start time [s]	Rise time [s]	t_Vmax1_ [s]	t_Vmax2_ [s]	Shrinkage after 24 h [%]	Density [kg/m^3^]
P_0	19.0 ± 1.5	52.5 ± 4.6	26.7 ± 1.8	30.9 ± 2.6	1.4 ± 0.2	33.54
P_200_10	24.8 ± 2.3	63.8 ± 5.1	35.8 ± 3.6	41.0 ± 4.1	0.8 ± 0.2	32.09
P_200_20	31.0 ± 4.3	79.0 ± 6.6	46.1 ± 5.9	52.4 ± 4.6	1.0 ± 0.2	32.83
P_200_30	48.6 ± 2.0	111.3 ± 7.7	70.2 ± 3.7	78.9 ± 6.0	1.2 ± 0.3	32.12
P_400_10	23.7 ± 2.3	59.3 ± 1.7	36.2 ± 2.6	38.6 ± 2.7	1.2 ± 0.1	32.47
P_400_20	31.8 ± 5.0	63.8 ± 8.5	42.8 ± 7.8	46.3 ± 7.5	1.2 ± 0.2	30.83
P_400_30	36.8 ± 4.6	73.3 ± 5.7	50.1 ± 4.2	53.3 ± 4.0	1.2 ± 0.1	30.57
P_600_10	19.3 ± 1.1	51.2 ± 2.5	28.0 ± 0.7	30.9 ± 1.8	1.1 ± 0.1	32.76
P_600_20	25.5 ± 4.1	56.3 ± 5.1	35.5 ± 3.7	38.4 ± 3.2	1.3 ± 0.1	31.97
P_600_30	27.9 ± 0.4	60.9 ± 3.4	39.4 ± 1.6	42.5 ± 1.5	1.2 ± 0.2	30.13

Comparing reference sample with samples with bio-based polyol, an increase of rise start time and rise time caused by the addition of can be observed. The most probable explanation of this effect is possible steric hindrances, the presence of less reactive secondary hydroxyl groups in bio-polyol structure, and the mildly acidic pH of bio-polyol which may reduce the activity of used catalysts^32^. Therefore, the rise start time and rise time are longer for systems with the addition of the most acidic polyol (P_200) and increase with the greater addition of each polyol. Moreover, a possible minor impact on these parameters may have the average molecular weight of polyols and hydroxyl value. Polyols synthesized with lower molecular mass PEGs may have a higher degree of branching, which causes more difficult spherical hindrances to overcome. Analyzing the course of the curves shown in Fig. 1, two maximal rising speeds (t_Vmax1_ and t_Vmax2_) can be noticed. This peak may be assigned to the activation of the catalysts at different temperatures during the exothermal reaction of substrates. Similarly, as for the rise start time and rise time, a significant delay of t_Vmax1_ and t_Vmax2_ is observed. It should be emphasized that the temperature difference between t_Vmax1_ and t_Vmax2_ has not changed noticeably. Moreover, one of possible explanation way of two-step growth process is assigning probable reactions to both steps. The first step of the growing stage may be assigned to the reaction between polyols and isocyanates and the second one to the thermally activated reaction of trimerization of isocyanates to polyisocyanurates^33^. Both reactions are exothermic, and the temperature increase associated with these reactions results in accelerated foam growth^34^. A surprising effect was noticed for the density of samples. As noted in the literature the growing viscosity of polyol mixture may cause problems during the formation of the cellular structure of foam and increase of viscosity. These difficulties should be more visible for foams obtained with more vicious polyols (e.g., P_600). The obtained results showed a decrease in foam density with the addition of bio-polyols, which may be explained by the presence of water particles in bio-based polyols. Residual water reacts with excess of isocyanate, what leads to the generation of CO_2_ particles and an additional foaming process. For this reason, to allow easier comparison of bio-polyols impact on foam properties, the amount of foaming agent (Solkane® 365/227) was modified to obtain samples with comparable densities. Overall, these results suggest that in the case of this PU system, bio-based polyol addition has a significant influence on the foaming process of PU foams. Comparing reference sample and samples with bio-polyol addition, the rise start time and the rise time were significantly increased and t_Vmax1_, and t_Vmax2_ have been delayed. This is mainly due to the reduced reactivity of bio-polyols due to their specific structure and pH. Slightly less important, but still significant, is the type of bio-polyol and its structure.

### Microstructure of bio-based foams (Scanning electron microscopy)

**Figure 2:**
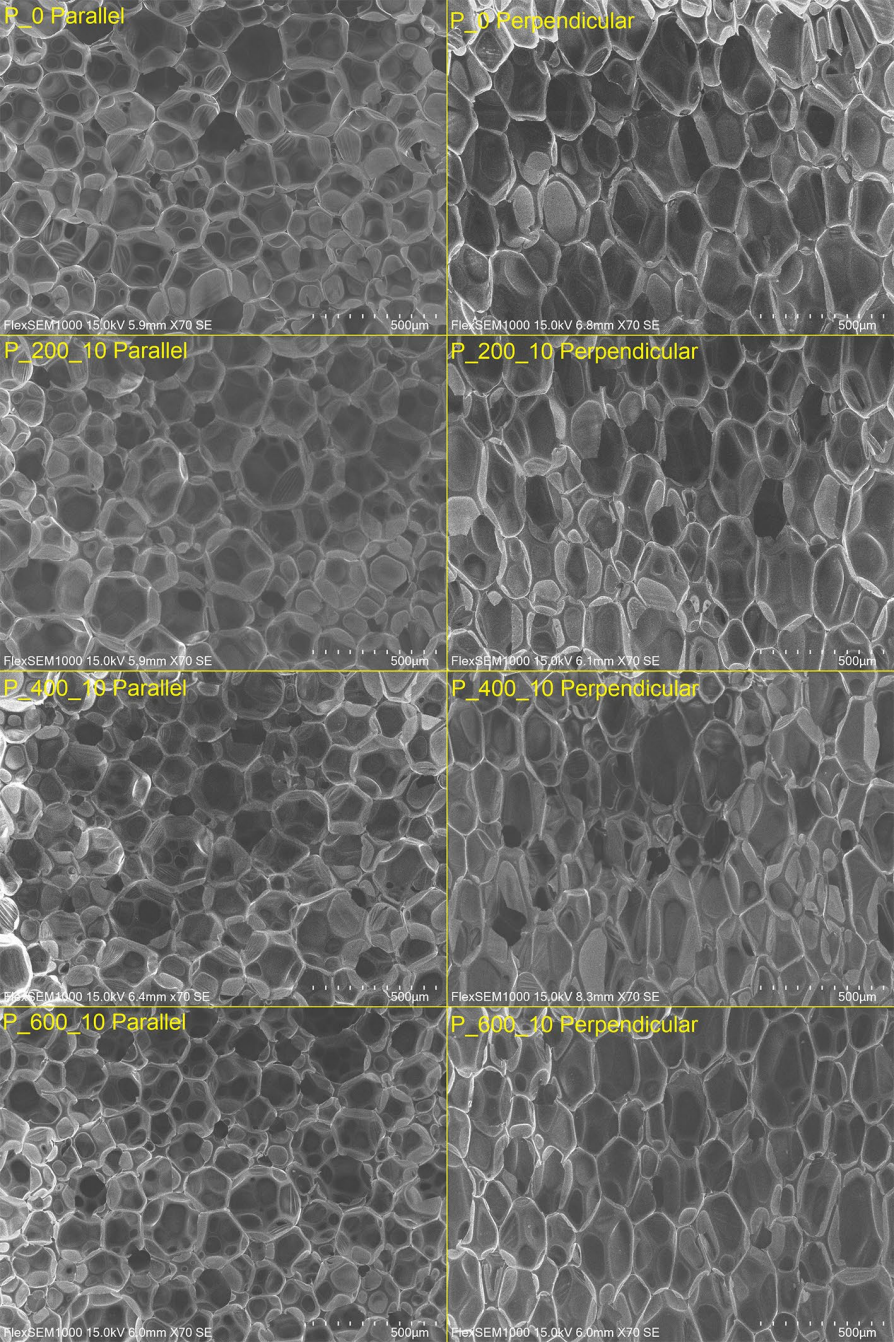
The microstructure of manufactured foams.

Figure 2 shows SEM images of selected manufactured foams in two directions (parallel and perpendicular to the foam rise). The structure of foams was analyzed and the calculated parameters of the structure are presented in Table 4. Analyzing the structure of the foams in both directions, an anisotropic structure which is significantly affected by bio-based polyol addition can be noticed. In the parallel direction, the addition of bio-based polyol caused an increase in average pore size. Additionally, average pore size increases with greater addition of bio-based polyol. A slight increase in the aspect ratio (ratio of the cell width to height), circularity and roundness with the addition of bio-based polyol can be observed. This may be due to the presence of water in bio-polyol, which causes the formation of circular pores. The aspect ratio of foams structure varies between 0.78 and 0.83, and has high circularity and roundness. For this reason, the resulting structure can be considered as circular to some extent.

**Table 4:** Parameters of cellular structure of manufactured polyurethane foams.

Direction	═ Parallel to the direction of growth ═									
Foam symbol	P_0	P_200_10	P_200_20	P_200_30	P_400_10	P_400_20	P_400_30	P_600_10	P_600_20	P_600_30
Average pore size [mm^2^]	0.027 ± 0.011	0.038 ± 0.012	0.043 ± 0.021	0.049 ± 0.020	0.040 ± 0.017	0.042 ± 0.014	0.044 ± 0.012	0.032 ± 0.013	0.033 ± 0.090	0.037 ± 0.015
Ferret diameter [μm]	219 ± 38	245 ± 40	256 ± 69	274 ± 55	244 ± 47	254 ± 41	262 ± 29	222 ± 37	228 ± 30	240 ± 52
Aspect ratio	0.779 ± 0.073	0.832 ± 0054	0.811 ± 0.067	0.823 ± 0.084	0.836 ± 0.073	0.823 ± 0.054	0.840 ± 0.076	0.817 ± 0.079	0.818 ± 0.052	0.825 ± 0.064
Circularity	0.859 ± 0.054	0.894 ± 0.033	0.899 ± 0.044	0.872 ± 0.055	0.905 ± 0.038	0.892 ± 0.044	0.890 ± 0.040	0.878 ± 0.053	0.865 ± 0.076	0.893 ± 0.041
Roundness	0.802 ± 0.098	0.864 ± 0.074	0.828 ± 0.076	0.846 ± 0.097	0.862 ± 0.038	0.855 ± 0.070	0.878 ± 0.089	0.847 ± 0.089	0.850 ± 0.064	0.849 ± 0.083
Direction	┴ Perpendicular to the direction of growth ┴									
Sample	P_0	P_200_10	P_200_20	P_200_30	P_400_10	P_400_20	P_400_30	P_600_10	P_600_20	P_600_30
Average pore size [mm^2^]	0.056 ± 0.025	0.047 ± 0.018	0.049 ± 0.015	0.051 ± 0.021	0.046 ± 0.017	0.051 ± 0.017	0.054 ± 0.021	0.045 ± 0.023	0.048 ± 0.011	0.053 ± 0.015
Ferret diameter [mm]	370 ± 88	312 ± 72	321 ± 53	327 ± 76	335 ± 69	367 ± 68	374 ± 83	301 ± 76	349 ± 46	360 ± 54
Aspect ratio [-]	0.531 ± 0.069	0.618 ± 0.086	0.622 ± 0.047	0.604 ± 0.052	0.526 ± 0.083	0.471 ± 0.049	0.499 ± 0.072	0.613 ± 0.071	0.506 ± 0.054	0.545 ± 0.051
Circularity [-]	0.766 ± 0.064	0.831 ± 0.054	0.837 ± 0.045	0.836 ± 0.038	0.776 ± 0.082	0.719 ± 0.056	0.741 ± 0.070	0.828 ± 0.041	0.758 ± 0.062	0.794 ± 0.048
Roundness [-]	0.531 ± 0.078	0.620 ± 0.091	0.625 ± 0.057	0.609 ± 0.096	0.526 ± 0.091	0.464 ± 0.054	0.490 ± 0.080	0.616 ± 0.074	0.505 ± 0.058	0.549 ± 0.058

Analyzing the foams structure in the perpendicular direction, a minor reduction of average pore size with the addition of bio-based polyol is observed. Additionally, the cell size increases with a greater addition of synthesized polyols and approaches the value of unmodified foam (P_0). This effect may be caused by the almost counterbalancing impact of the water foaming effect (smaller and circular cells), and the increase of viscosity which hinders the foaming process of PU. Moreover, significant cell elongation in a perpendicular direction can be noticed, which is a common effect caused by the foaming process in the dimensionally constrained mold^35^. The aspect ratio of foams in perpendicular direction varies between 0.47 and 0.62, and has significantly lower circularity and roundness in comparison to parallel direction. Additionally, the differences between the cell structure of each foam in a perpendicular direction are more visible. Surprisingly, the addition of bio-polyol P_200 caused an increase in aspect ratio, circularity and roundness of the pores structure. One of the possible explanations of this effect may be the presence of water, differences in viscosity of bio-based polyols and in the foaming process which were discussed above. On the other hand, the addition of a more vicious polyol with a comparable amount of water caused increased anisotropy of cell structure. This phenomenon emphasizes the influence of the molar mass of polyols and viscosity on the foaming behavior of polyurethane materials.

### Open cell content and thermal conductivity of manufactured foams

Thermal conductivity coefficient (*λ*) is a key parameter which determines the application of cellular materials as insulation of buildings and electrical equipment. The results of thermal conductivity, open cell content and density measurements are presented in Table 5. An extremely important properties that affect thermal conductivity are density and open cell content. All manufactured foams have a density of 32 ± 2 kg/m^3^ therefore the impact of this parameter on λ was limited. Analyzing the thermal conductivity of manufactured samples slight increase of *λ* with the addition of bio-based polyol is observed. This effect is likely caused by the addition of a bio-polyol with a different viscosity and reactivity, which disrupted the foaming process. For this reason, the average size of pores increased and more open cells were formed, which presence accelerates heat transport^36^. This effect is especially visible for samples with 30% bio-polyols content. Similar results were observed by Gama et al.^37^ where the addition of bio-based polyol from liquefied coffee grounds caused a slight increase in open cell content. Analyzing the effect of molecular mass of bio-polyols on foam properties, there is no significant difference between the thermal conductivity of samples. This may be the evidence that the chemical structure of manufactured foams is a minor factor but the most important effect on thermal conductivity has the cellular structure of the sample. For this reason, the thermal conductivity of P_200 samples with higher bio-polyol addition are slightly lower due to lower open cell content which may be caused by a more regular and longer foaming process.

**Table 5:** Thermal conductivity, open cell content and density of manufactured foams.

Foam symbol	λ [mW/m·K]	Open cell content [%]	Density [kg/m^3^]
P_0	21.01 ± 0.12	13.41 ± 0.46	33.54
P_200_10	22.33 ± 0.20	15.94 ± 0.30	32.09
P_200_20	22.24 ± 0.66	15.61 ± 1.09	32.83
P_200_30	21.96 ± 0.74	17.21 ± 0.47	32.12
P_400_10	21.98 ± 0.18	14.18 ± 0.60	32.47
P_400_20	22.98 ± 0.30	17.27 ± 0.61	30.83
P_400_30	25.21 ± 0.66	19.55 ± 0.54	30.57
P_600_10	22.08 ± 0.95	17.91 ± 0.15	32.76
P_600_20	22.14 ± 0.40	18.27 ± 1.25	31.97
P_600_30	24.04 ± 0.86	19.39 ± 0.09	30.13

### Mechanical properties (Tensile and compression tests)

**Figure 3:**
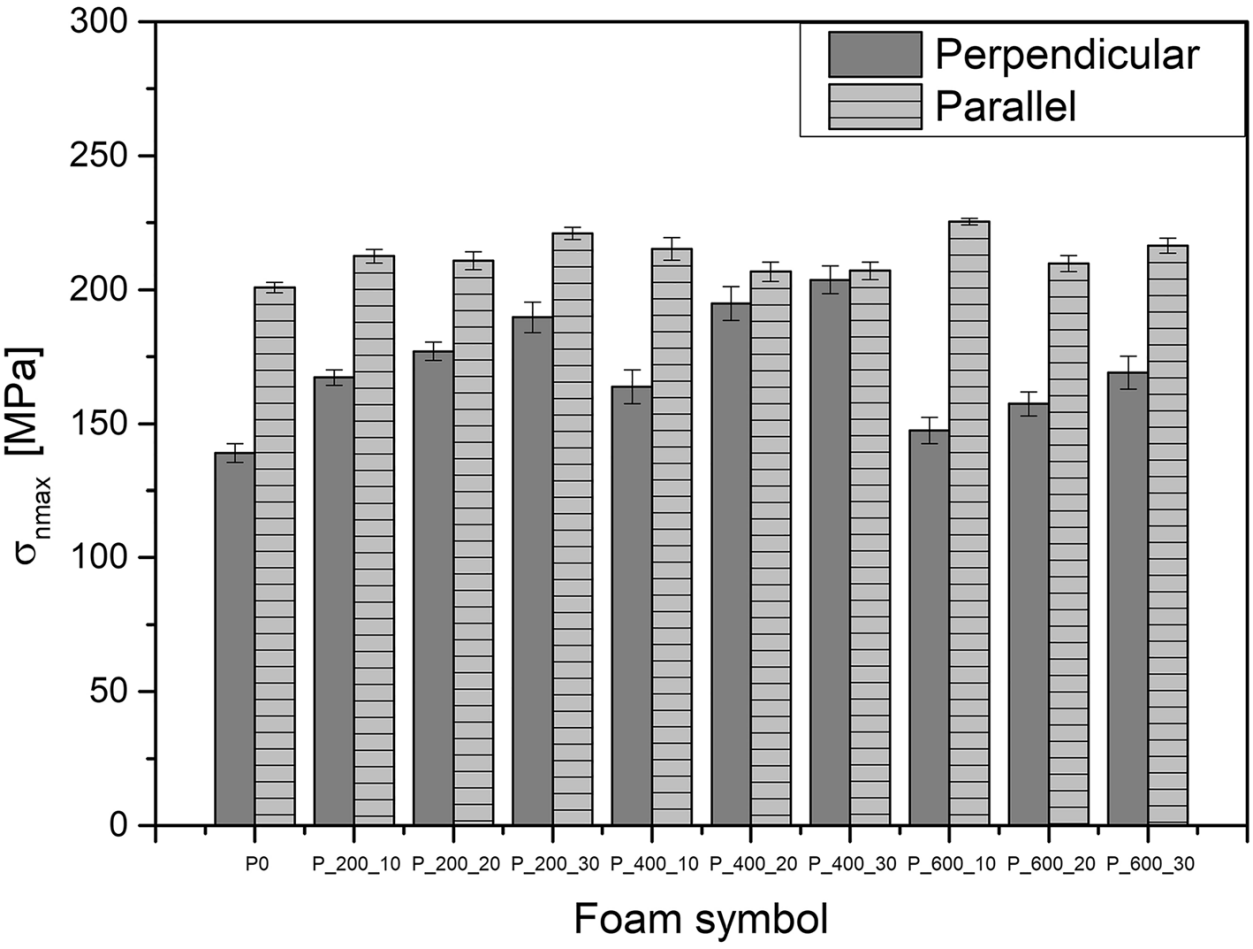
Results of uniaxial compression tests of manufactured foams.

The mechanical properties of manufactured foams are presented in Fig. 3 and Table 6. As the density and anisotropy of cell structure have a significant influence on the mechanical properties of polyurethane foams, uniaxial compression tests were conducted for samples cut in perpendicular and parallel directions. Moreover, to precisely compare the effect of bio-polyol addition on PU foams properties, normalized compression strength (σ_nmax_) was calculated^38^. Analyzing obtained values of normalized compression strength in the perpendicular direction, a significant increase of this parameter with a greater addition of bio-polyols can be observed. This effect may be caused by the increase in the degree of crosslinking due to the addition of the bio-polyols with higher hydroxyl value, and functionality^37,39,40^. The strengthening effect was stronger for samples with the addition of bio-polyol with lower molecular masses, especially for P_200 samples, which contain shorter and less flexible PEG 200 chains. For this reason, P_600 samples have slightly lower compression strength than other samples. Moreover, possible water reaction with isocyanates caused the formation of stiffer and more durable structures (e.g., urea’s, burets) which may increase crosslinking density and compressive strength^41^. Additionally, differences in the foaming process modified the cellular structure of PU materials, which influences load transfer^40^. In the perpendicular direction, there is no significant corelation between the aspect ratio and compressive strength. In the case of this material, we believe that the chemical structure of the material had a critical influence on the results of uniaxial compression tests. For this reason, compressive strength increases with the amount of bio-polyol. On the other hand, the increase of compressive strength in the parallel direction is almost unnoticeable. This may be caused by the balancing effect of reinforcement of the polyurethane network (increase of compression strength) and modification of cellular structure which limits load transfer in the parallel direction (higher aspect ratio).

**Table 6:** Results of uniaxial tensile testing of manufactured foams.

Foam symbol	Tensile Strength [kPa]	Maximal deformation [%]	Young modulus [MPa]
┴			
P0	258 ± 27	9.0 ± 1.1	3.55 ± 0.26
P_200_10	241 ± 7	9.9 ± 1.0	4.54 ± 0.43
P_200_20	244 ± 12	9.4 ± 1.1	3.98 ± 0.57
P_200_30	261 ± 27	9.8 ± 1.5	4.26 ± 1.40
P_400_10	246 ± 18	9.2 ± 1.1	4.55 ± 1.82
P_400_20	222 ± 14	9.4 ± 1.1	3.76 ± 0.93
P_400_30	238 ± 29	9.2 ± 1.1	4.81 ± 0.60
P_600_10	243 ± 11	9.8 ± 1.1	3.90 ± 0.98
P_600_20	226 ± 15	8.6 ± 0.4	3.91 ± 0.23
P_600_30	223 ± 25	7.6 ± 1.0	4.70 ± 0.64

Analyzing the results of uniaxial tensile tests in the perpendicular direction (tests in the parallel direction were not carried out due to the dimensional limitations of the samples) slight decrease in tensile strength can be noticed. This decrease may be caused by the increase in the brittleness of PU foams caused by the presence of water in polyols^42^. Moreover, polyols synthesized through liquefaction may not act as conventional soft segments, which determines PU elasticity^43^. Despite, the fact of addition of bio-polyols which results in the increase in crosslinking density and compression strength, the decisive influence on tensile properties has increase in the brittleness, which causes accelerated fracture of the PU network. Analyzing the influence of bio-polyols molecular weight, it can be noticed that P_200 samples caused a lower decrease in the tensile strength which is in line with the previously described tests and is associated with a potentially more stable structure of samples.

### Thermal properties (Thermal degradation, thermal conductivity coefficient, cone calorimeter)

**Figure 4:**
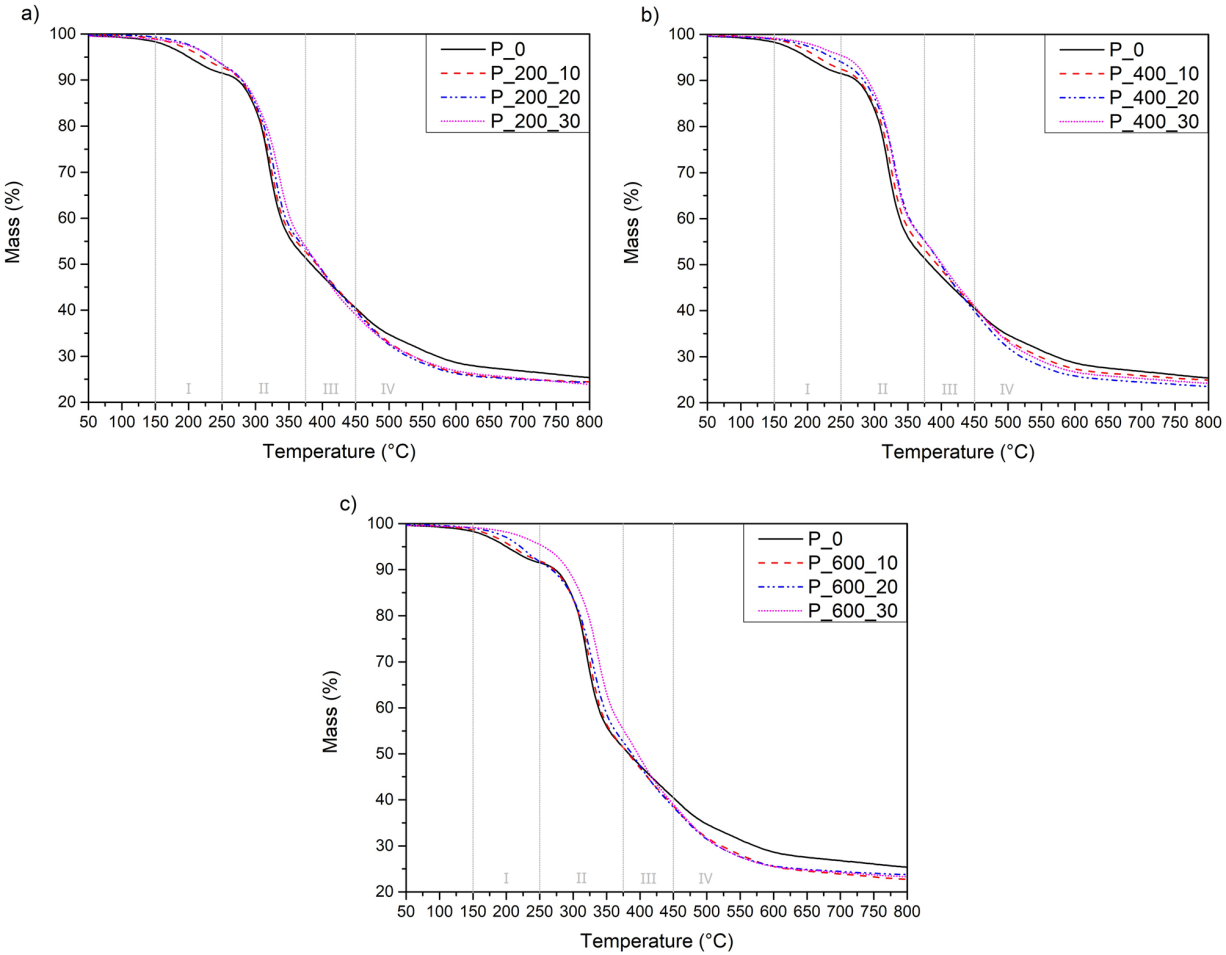
Thermogravimetric (TG) curves of foams with addition of bio-based polyols; (**a**) P_200; (**b)** P_400; (**c**) P_600.

**Figure 5:**
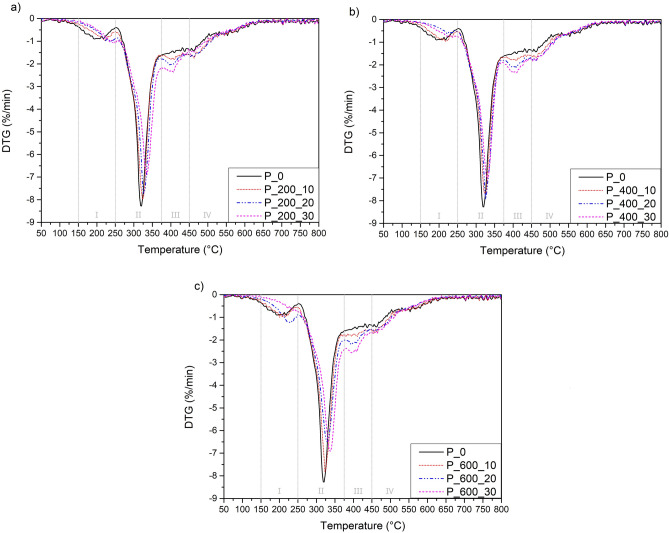
DTG curves of curves of foams with addition of bio-based polyols; (**a**) P_200; (**b**) P_400; (**c**) P_600.

To determine the thermal stability of manufactured samples, thermogravimetric analysis (TGA) was conducted. The results of TGA are presented in Figs. 4, 5 and Table 7. Analyzing the obtained curves, it can be noticed that the addition of bio-based polyols with different molecular weights has a noticeable impact on the thermal degradation process of manufactured materials. On the other hand, it should be noted that the difference in thermal stability between samples with the addition of different polyols is not significant. The thermal stability of bio-based polyols was determined during our previous studies^31^.

**Table 7:** Results of thermogravimetric analysis of manufactured polyurethane foams.

Foam symbol	% Mass loss temperature [°C]				T_max1_ [°C]	Char residue [%]
	T_2%_	T_5%_	T_10%_	T_50%_		
P_0	157.5	200.0	273.3	383.4	319.6	25.39
P_200_10	178.0	218.6	277.0	400.2	324.8	24.56
P_200_20	182.3	234.5	284.8	394.1	331.0	24.05
P_200_30	187.2	234.4	280,4	392.4	335.4	23.88
P_400_10	175.7	219.9	277.2	394.1	324.4	24,92
P_400_20	188.2	237.0	284.3	399.6	330.4	23.54
P_400_30	201.2	257.7	290.2	401.5	325.8	24.24
P_600_10	167.4	208.4	271.4	382.5	323.8	22.71
P_600_20	177.2	222.8	270.5	388.7	333.8	23.82
P_600_30	205.2	255.7	292.2	396.6	337.4	23.30

In this study, the addition of bio-based polyols not only changed the thermal stability of manufactured materials but also modified the process of degradation. All obtained curves present a four-step degradation process, with one significant temperature of maximal degradation. The thermal degradation process of manufactured foams is complicated and complex process due to the composition of PU foams which consist of eleven different components. The first step of degradation which occurs in the temperature range of 150–250 °C may correspond to the degradation of flame retardant TCCP (temperature of decomposition T = 244 °C), degradation of biuret and allophanate groups which has lower thermal stability^44^ and partial degradation of PU bio-polyol segments in PU which are composed of glycerol and glycerol derivates^45^. This stage of degradation corresponds to temperatures of 2% (T_2%_) and 5% (T_5%_) mass degradation. T_5%_ which is commonly recognized as the beginning of the thermal decomposition process, increased from 200.0 °C for P_0, to 257.7 °C for the P_400_30 sample. Moreover, T_10%_ shifted from 273.3 °C for P_0 to 290.0 °C for P_400_30 sample. An increase in both temperatures may indicate an increase in thermal stability by the implementation of bio-polyols.

The second degradation step which occurs at a temperature range from 250 to 375 °C with a noticeable maximum around 325 °C may be related to the degradation of foam rigid segment composed of isocyanates, urethanes and isocyanurate groups to lower molecular weight components, amines and polyols^46^. Analyzing the maximal degradation temperature, a significant shift from 319.6 to 337.4 °C with the addition of bio-based polyol can be noticed. This shift with greater bio-polyol addition may be due to an increase in crosslinking degree and stabilization of the foams structure caused by the addition of more thermally stable bio-based polyol. The third degradation step which occurs in the range from 375 to 450 °C may be connected to the degradation of soft segments which are composed of petrochemical polyol (ELAPOL) and polyethylene glycol (PEG) parts of bio-based polyol^47^. The degradation of polyethylene glycol (PEG) parts of bio-based polyol was confirmed in our previous studies^31^, where thermogravimetric analysis showed a peak of intensive polyol degradation at around 375–392 °C. At temperatures above 450 °C the peak overlapping with the peak of the third degradation step can be noticed and may be due to the thermolysis of degradation products formed during previous degradation steps.

### Evaluation of flammability of manufactured foams

**Figure 6:**
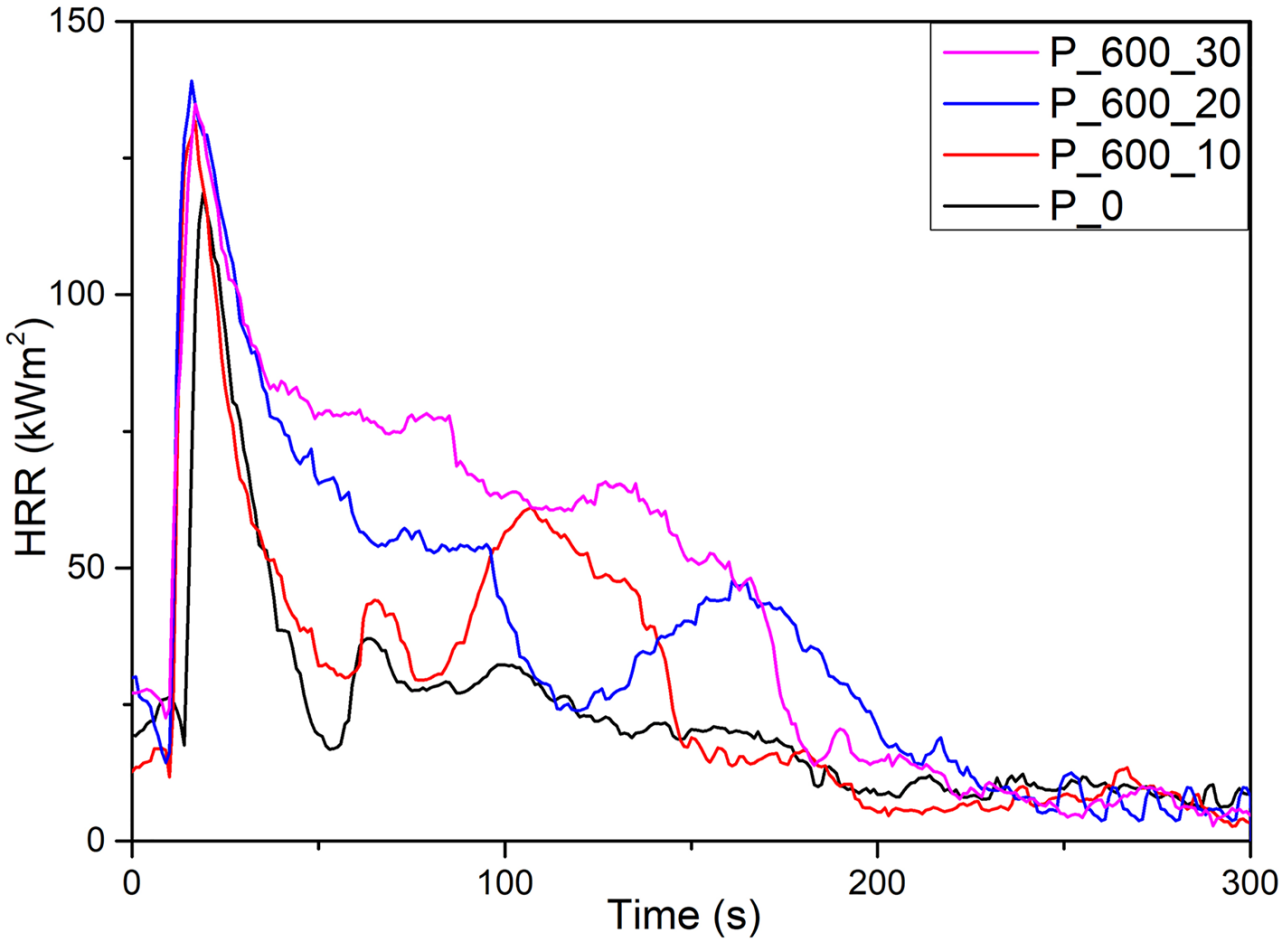
Examples of HHR curves for PUR-PIR foams synthesized with addition of P_600 polyol.

To determine the impact of bio-polyols addition on the combustion process of industrially applied PUR-PIR foams, cone calorimetry was conducted. The results of this test are presented in Tables 8 and 9. Additionally, selected heat release rate (HRR) curves are presented in Fig. 6. The tests revealed no significant difference between time to ignition (TTI) and time to flameout (TTF) of manufactured foams. On the other hand, the test showed a significant increase in total heat release with an increasing amount of synthesized bio-based polyols. In comparison to reference foam (P_0), THR increased by 22%, 71% and 74% for P_200_10, P_200_20, and P_200_30 samples, respectively. Analyzing curves presented in Fig. 6 sharp increase of HRR until maximal peak of heat release rate (PHRR) was observed. There are no significant differences between the pHRR of samples. For reference sample (P_0) after pHRR, heat release rate decreases remain at a low level. This may indicate the formation of a protective char layer which prevents further degradation^48^. For P_600_10 second peak can be noticed, which may be evidence of char layer damage and further degradation of the sample and decomposition of intermediary degradation products or intermediary char^49^. For samples with greater addition of bio-polyols intensive degradation after pHRR can be noticed. This indicates the difficulties in forming of stable char layer on the material surface and the continuous degradation of the sample till flameout. For this reason, THR increases with bio-polyols greater addition. This may indicate the disturbed formation of the char layer due to the presence of bio-polyols, which degradation products may damage char layers. For these reasons, samples with bio-polyol release significantly more heat than reference sample. This is reflected in the increased average value of HRR and the maximum average rate of heat emission (MAHRE). Moreover, this effect may be enhanced by a small decrease in the amount of TCPP which is flame retardant and acts in the gas phase. During the decomposition of TCPP, PO and Cl radicals are generated. These radicals may interfere with the combustion process by reduction of the flame energy.

**Table 8:** Selected parameters of combustion process of PUR-PIR foams.

Foam symbol	TTI [s]	TTF [s]	THR [MJ/m^2^]	pHRR [kW/m^2^]	AvHRR [kW/m^2^]	MARHE [kW/m^2^]
P_0	5.3 ± 0.6	221 ± 48	6.3 ± 0.6	127.8 ± 8.1	20.8 ± 1.8	63.45 ± 9.83
P_200_10	4.3 ± 0.7	245 ± 37	7.7 ± 0.2	136.1 ± 7.8	26.3 ± 1.7	74.90 ± 5.88
P_200_20	4.0 ± 0.5	237 ± 13	10.8 ± 0.3	135.0 ± 7.0	36.0 ± 1.0	82.05 ± 11.67
P_200_30	3.7 ± 1.2	205 ± 14	11.0 ± 0.4	142.5 ± 3.3	36.9 ± 1.3	82.00 ± 4.81
P_400_10	4.3 ± 0.6	255 ± 31	9.0 ± 0.3	127.6 ± 4.6	29.2 ± 1.1	68.13 ± 2.05
P_400_20	4.3 ± 1.5	215 ± 32	10.7 ± 1.2	129.6 ± 3.7	35.7 ± 3.8	77.20 ± 2.97
P_400_30	4.7 ± 1.5	240 ± 26	11.2 ± 0.4	121.1 ± 5.6	37.7 ± 0.8	75.63 ± 2.85
P_600_10	5.0 ± 0.5	252 ± 20	7.7 ± 0.4	132.3 ± 5.1	25.7 ± 0.5	67.85 ± 3.46
P_600_20	4.7 ± 0.6	232 ± 38	10.4 ± 0.5	131.5 ± 8.3	34.6 ± 1.6	72.70 ± 1.71
P_600_30	4.3 ± 0.6	209 ± 13	11.7 ± 0.6	128.8 ± 3.1	38.8 ± 2.1	73.25 ± 1.63

*TTI* time to ignition, *TTF* time to flameout, *THR* total heat release, *pHRR* maximal peak of heat release rate, *AvHRR* average heat release rate, *MAHRE* maximum average rate of heat emission.

**Table 9:** Selected parameters of smoke emission during combustion of PUR-PIR foams.

Foam Symbol	TSR [m_2_/m_2_]	TSP [m^2^]	AvCO [kg/kg]	AvCO_2_ [kg/kg]	AvCO/AvCO_2_ Weight Ratio
P0	170.9 ± 13.6	1.50 ± 0.10	1.22 ± 0.06	3.56 ± 0.19	0.34 ± 0.04
P_200_10	257.8 ± 19.9	2.27 ± 0.21	1.27 ± 0.02	3.67 ± 0.21	0.35 ± 0.02
P_200_20	313.5 ± 4.1	2.77 ± 0.06	1.03 ± 0.10	4.25 ± 0.12	0.24 ± 0.03
P_200_30	411.8 ± 12.3	3.67 ± 0.12	1.08 ± 0.02	4.03 ± 0.15	0.27 ± 0.01
P_400_10	283.2 ± 33.9	2.53 ± 0.31	1.24 ± 0.09	3.84 ± 0.02	0.32 ± 0.02
P_400_20	332.9 ± 29.3	3,00 ± 0.36	1.22 ± 0.12	4.39 ± 0.21	0.28 ± 0.04
P_400_30	385.0 ± 22.0	3.40 ± 0.20	1.15 ± 0.12	4.19 ± 0.33	0.27 ± 0.02
P_600_10	235.3 ± 2.5	2.10 ± 0.14	1.19 ± 0.07	3.80 ± 0.13	0.31 ± 0.03
P_600_20	487.2 ± 34.4	4.33 ± 0.32	1.16 ± 0.13	4.11 ± 0.19	0.28 ± 0.04
P_600_30	394.4 ± 24.8	3.47 ± 0.23	1.23 ± 0.08	4.06 ± 0.06	0.30 ± 0.02

*TSR* total smoke release, *TSP* total smoke production, *AvCO* average CO emission, *AvCO*_*2*_ average CO_2_ emission.

From Table 9, it can be noted that the addition of bio-polyols significantly increased the total smoke release (TSR) and total smoke production (TSP) of manufactured foams. An increase of these parameters may be caused by the longer process of burning out of samples which may be caused by above mentioned reduction of char layer stability. Increased production of smoke during combustion may cause possible danger during fire, as increased smoke can reduce visibility during evacuation and may cause smoke poisoning after inhalation^48^. Increased smoke production did not affect on generated amount of carbon monoxide (CO), which causes a high risk when breathing (possibility of asphyxiation). On the other hand, the generated amount of carbon dioxide increases with the addition of bio-based polyol may also be dangerous for human health. This value is higher for samples with a higher addition of bio-based polyol. Moreover, TCPP is recognized as a substance which may increase the amount of generated gases. In this case, samples with higher TCCP content show comparable or even lower TSR, TSP, CO and CO_2_ emissions. It may be caused by limited thermal degradation of samples. It should also be noted that CO_2_ and CO are not the only gases that can be released during the combustion of polyurethane materials. In order to assess the potential harmfulness of the gases, it would be necessary to conduct a much more thorough qualitative and quantitative analysis. Overall, these results indicate that the flammability of manufactured materials was slightly deteriorated by the addition of bio-based polyols. The increase in flammability should be balanced by the addition of suitable flame retardants. One of possible solution may be to increase of the amount of TCCP in the system. This may result in the reduction of foams mechanical properties because TCCP penetrates between the polymer chains and acts as a plasticizer. Other solutions may include the addition of expandable graphite, aluminum hydroxides or montmorillonite clays. These substances will reduce the flammability of the system and increase the mechanical properties, but may also increase the thermal conductivity of foams, modification of the foaming process and price of foam.

## Conclusions

In summary, previously synthesized bio-polyols with different chain lengths via solvothermal liquefaction were used as a substitute for petrochemical polyols in the manufacturing of polyurethane-polyisocyanurate foams. Ten sets of PUR-PIR foams were manufactured with the addition of up to 30% of bio-based polyols. The influence of polyols with different molecular weights on the foaming process and properties of foams was examined by FOAMAT device, mechanical testing, thermogravimetric analysis, cone calorimetry, scanning electron microscopy and other tests. It should be noted that the biggest impact on properties has the addition of bio-polyols, but their molecular weight is a less significant parameter.

The conducted analysis showed the significant impact of bio-polyol on the foaming process of PUR-PIR foams. The extension of foam growth time by up to 112% may be explained by possible steric hindrances, the presence of less reactive secondary hydroxyl groups, and the mildly acidic pH of the bio-polyol. Moreover, the addition of bio-polyols with reduced reactivity and different viscosity modified the cell growth process, which resulted in a change in the average pore size and aspect ratio of cells. Analysis of mechanical properties has shown an increase in compressive strength from 139 kPa to 203.67 for P_0 and P_400_300 samples, respectively. This can be assigned to the increase of cross-linked by the addition of bio-based polyols with higher hydroxyl value and functionality. Thermogravimetric analysis has shown a four-step degradation process of manufactured foam where T_5%_ increases with the addition of bio-polyols with higher molecular mass. T_2%_ increased by 57.7 °C for the P_400_30 sample in comparison to reference foam. Investigation of the combustion process of manufactured foams by cone calorimetry has shown an increase in heat release rate, total heat release and amount of produced smoke with increasing amounts of bio-polyols. This may be caused by difficulties in forming of stable char layer on the material surface. These results indicate that the flammability of manufactured materials was slightly deteriorated by the addition of bio-based polyols.

Overall, this paper provides new insights into the manufacturing of foamed materials with the addition of bio-polyols with different chain lengths and structures. Moreover, the results of this study underline the importance of the replacement of petrochemical resources by more sustainable components. Further research is needed to assess the environmental impact of foam modification by bio-based polyols and investigate the composition of the emitted gases during foam combustion.

## Data Availability

Data available on request from the authors.
